# Neuropeptides Control the Dynamic Behavior of Airway Mucosal Dendritic Cells

**DOI:** 10.1371/journal.pone.0045951

**Published:** 2012-09-26

**Authors:** Sabrina Voedisch, Sabine Rochlitzer, Tibor Z. Veres, Emma Spies, Armin Braun

**Affiliations:** 1 Department of Airway Immunology, Fraunhofer Institute for Toxicology and Experimental Medicine; Biomedical Research in Endstage and Obstructive Lung Disease Hannover (BREATH), Member of the German Center for Lung Research, Hannover, Germany; 2 Department of Immunology, Hannover Medical School (MHH), Hannover, Germany; 3 University of Turku, MediCity Research Laboratory, Turku, Finland; Leiden University Medical Center, The Netherlands

## Abstract

The airway mucosal epithelium is permanently exposed to airborne particles. A network of immune cells patrols at this interface to the environment. The interplay of immune cells is orchestrated by different mediators. In the current study we investigated the impact of neuronal signals on key functions of dendritic cells (DC). Using two-photon microscopic time-lapse analysis of living lung sections from CD11c-EYFP transgenic mice we studied the influence of neuropeptides on airway DC motility. Additionally, using a confocal microscopic approach, the phagocytotic capacity of CD11c^+^ cells after neuropeptide stimulation was determined. Electrical field stimulation (EFS) leads to an unspecific release of neuropeptides from nerves. After EFS and treatment with the neuropeptides vasoactive intestinal peptide (VIP) or calcitonin gene-related peptide (CGRP), airway DC in living lung slices showed an altered motility. Furthermore, the EFS-mediated effect could partially be blocked by pre-treatment with the receptor antagonist CGRP_8–37_. Additionally, the phagocytotic capacity of bone marrow-derived and whole lung CD11c^+^ cells could be inhibited by neuropeptides CGRP, VIP, and Substance P. We then cross-linked these data with the *in vivo* situation by analyzing DC motility in two different OVA asthma models. Both in the acute and prolonged OVA asthma model altered neuropeptide amounts and DC motility in the airways could be measured. In summary, our data suggest that neuropeptides modulate key features motility and phagocytosis of mouse airway DC. Therefore altered neuropeptide levels in airways during allergic inflammation have impact on regulation of airway immune mechanisms and therefore might contribute to the pathophysiology of asthma.

## Introduction

The airways are permanently exposed to environmental stimuli such as temperature and humidity shifts, airborne pathogens, pollen and smoke particles, or ozone. To enable homeostasis of lung physiology several mechanisms exist to compensate this blast of influences. Down the cascade of defense mechanisms (mucociliary elevator, IgA, epithelial barrier), a multitude of immune cells patrols below the epithelial layer to intercept foreign particles and antigens. Mostly macrophages and dendritic cells (DC) capture, process and present incoming antigen and initiate appropriate immune responses. Nerves co-localizing with DC below the epithelial layer respond to chemical, mechanical or inflammatory stimuli and on their part interact with the surrounding cells via neurotransmitters and neuropeptides. DC and other immune cells can receive these neurogenic signals by expressing neuropeptide receptors [1]–[4].

Neuropeptides that do not belong to classical transmitters of the parasympathetic or sympathetic nervous system are classified under the term non-adrenergic non-cholinergic (NANC) peptides. Examples for such mediators are calcitonin gene-related peptide (CGRP), substance P (SP) (excitatory NANC), and vasoactive intestinal peptide (VIP) (inhibitory NANC). These neuropeptides can act in general on bronchus and capillary muscle tone, secretion, and immune cells. Activation of sensory neurons upon stimuli leads via axon reflex mechanisms to the release of SP and CGRP in the airways [5]. SP has been reported to lead to the release of pro-inflammatory cytokines (IL-6, IL-8, TNFα) from human bronchial epithelial cells. SP can also induce expression of the integrin ICAM-1 that is important for the recruitment of immune cells to the lung and it can promote the survival of DC [4], [6], [7]. VIP is an anti-inflammatory peptide [8], capable of inducing the generation of tolerogenic DC that in turn can induce regulatory T cells [9]. CGRP can reduce antigen presenting capacity of DC thereby affecting the outcome of allergic airway inflammation [3]. CGRP and VIP both can also act as chemoattractants on naïve DC [9].

Reports have been assuming that in respiratory diseases like asthma the interplay between nerves and immune cells is misbalanced. These alterations are encompassed under the term “neurogenic inflammation” [1], [10]–[12]. There are e.g. higher SP concentrations in broncho alveolar lavage fluid (BALF) of asthma patients that further increase with allergen challenge [13]. Moreover three to four times higher CGRP and SP expression could be observed 24 h after allergen challenge in guinea pig airway tissue [14]. Whereas one finds low plasma levels of VIP in human patients the levels for SP and CGRP are elevated [15].

In the sensitization phase, DC are important for Th2-differentiation of naïve CD4^+^ T cells specific to an aeroallergen [16]. During asthma, DC are also important to maintain eosinophilic airway inflammation by recruiting primed Th2 cells to the lung. In turn, Th2 cells are important key effectors producing cytokines like IL-4, IL-5, and IL-13 [6], [17], [18].

Here we investigated the interaction between the nerves and immune cells in mouse airways. We addressed the question whether neuropeptides can influence the behavior of a defined immune cell population in the airway compartment. We identified CD11c^+^ cells as airway mucosal DC based on various specific markers in addition to their anatomical localization and morphology. In living tissue we revealed the dynamics of this DC population under the influence of neuropeptides. As readout parameters we determined the motility and phagocytotic capacity of airway mucosal DC after different neuropeptide stimuli. Our data show that neuropeptides can modulate these key features of DC. By linking our findings with two different OVA-induced asthma models in mice, it appears that neuropeptides are involved in the adjustment of DC behavior during allergic airway inflammation.

## Methods

### Ethics Statement

All animal experiments were performed in concordance with the German animal protection law under a protocol approved by the appropriate governmental authority (*Niedersächsisches Landesamt für Verbraucherschutz und Lebensmittelsicherheit*).

### Mice

CD11c-EYFP-transgenic mice [19], CX_3_CR1*^gfp/+^*(20) (both C57BL/6 background), and wild-type C57BL/6 mice (purchased from Charles River, Sulzfeld, Germany) were used at an age of 10–12 weeks.

### Preparation of Living Lung Slices for Time- lapse Imaging

In order to prepare living lung slices, mice were euthanized with an overdose (300 mg/kg bw) of pentobarbital. Lungs of CD11c-EYFP mice were filled with 2 ml of liquid 2% low melting agarose until polymerized. Lungs were then cut into 300 µm thick sections using an OTS-5000 oscillating tissue slicer (Electron Microscopy Sciences, Hatfield, PA). To expose the airway mucosa for imaging, lungs were put in place allowing the production of slices with longitudinally cut airways. Lung sections were cultivated in a 37°C tempered, 5% carbon dioxide gassed incubator (Heraeus 6000, Hanau, Germany) until imaging. Time-lapse imaging was performed in a heated and medium sustentative imaging chamber [20].

### Electrical Field Stimulation

Electrical field was applied in the imaging chamber using an S48 stimulator (Warwick, RI) under the following conditions: train rate 1 train per second (TPS), 4 ms train duration, 20 Hz stimulation rate, 3 ms delay, 2 ms duration, 50 V twice for 1 min with 3 min brake. Samples were analyzed for 1 h or 24 h after electrical stimulation of living lung sections or magnetically sorted CD11c^+^ cells from whole lung tissue, cultured on collagen-covered glass slides. For neuropeptide receptor antagonist studies, slices were firstly incubated for 5 min with the antagonist CGRP_8–37_ (100 nM), were then electrically stimulated and analyzed after 1 h incubation. For one experiment we analyzed eight living lung slices (four control and four treated slices) originating from one individual mouse.

### Neuropeptide Stimulation

For neuropeptide treatments, living lung slices were prepared and cultivated overnight. *In vitro*-generated bone marrow-derived DC (BMDC) were cultured for 8–10 days and were then seeded overnight on collagen-covered glass slides (Advanced BioMatrix, San Diego, CA). Glass slides allowed a convenient transfer of cells into the imaging chamber. Neuropeptides (solved in water) or water as control were applied 5 min prior to imaging to the lung section/cultured cells under gentle shaking and were then removed by transferring the section/cultured cells into the imaging chamber with continuous medium perfusion. VIP was applied with a final concentration of 0.1 nM, SP with 1 µM, and CGRP with 1 nM (all from Phoenix pharmaceuticals, Burlingame, CA). The antagonist CGRP_8–37_ was used at a final concentration of 100 nM. For one experiment we analyzed eight living lung slices (four control and four treated slices) originating from one individual mouse.

### Two-photon Time-lapse Microscopy of Mouse Main Bronchi

Living lung sections were placed in an imaging chamber allowing continuous medium supply and a stable temperature of 37°C. Sections were kept in place with a slice anchor (Harvard Apparatus, Holliston, MA). Imaging time was 1 h per lung section. Control and treated sections were imaged alternately and at the same day to compare sections with similar time delay after tissue collection and at similar time points after treatment. Imaging was performed with a TrimScope two-photon microscope (LaVision BioTec, Bielefeld, Germany) attached to a Spectra-Physics Titanium Sapphire MaiTai HP ultrafast laser (Newport, Irvine, CA) using a 20× water immersion objective (NA 0.95). Second harmonic generation signals (SHG) of collagen fibers and enhanced yellow fluorescent protein (EYFP) fluorescence of CD11c-EYFP^+^ cells were excited with 920 nm (7% of 2.65 W output power) and detected via 490/50 or 525/50 band pass filters, respectively. To generate four-dimensional time-lapse images of moving DC each minute of one hour three dimensional *z*-stacks were acquired with a 1 µm *z*-resolution resulting in image volumes of 500 µm in *xy* and 30 to 60 µm in *z*-direction.

### Analysis of Cell Motility

Cell tracking and mean velocity determination was performed with Imaris software version 7.2.3 (Bitplane, Zürich, Switzerland), controlled manually and applying the following criteria. Objects with a diameter above 13 µm were considered to be cells, since this criterion turned out empirically to match best to identify living cells for analysis. Only tracks lasting longer than 300 sec and with a displacement above 2.5 µm were included for analysis. Spots not being located in the airway epithelium, i.e. not matching with the SHG signal, were excluded from analysis. Broken tracks were corrected manually via the spot connect option of the spot wizard. If drift correction was necessary, dead (round, immobile) cells were used as internal references to correct drift and were later excluded from analysis. Statistical analysis was performed by comparing each treated group with their according control group using GraphPad Prism 4.03 and applying Mann-Whitney test.

### Generation of Bone Marrow-derived Dendritic Cells

For the *in vitro* generation of DC from the bone marrow, standard protocols were implied (22). Shortly, mice were sacrificed; tibiae were separated, sterilized in ethanol, and flushed with medium to isolate bone marrow. Erythrocytes were removed by lysis. Remaining cells were cultivated with 20 ng/ml rmGM-CSF (Immunotools, Friesoythe, Germany) under standard conditions in a cell culture incubator. To determine the phagocytotic capacity, cells were harvested at day 9 or 10 and seeded into a 96-well plate. As a control in the phagocytosis assay (see below) a fraction of cells was triggered to mature with 100 ng/ml LPS from *E. coli* serotype 0111:B4 (Sigma, München, Germany) over night.

### *In vitro* Cultivation of Lung CD11c^+^ Cells

To culture lung CD11c^+^ cells *in vitro*, mice were euthanized with an overdose of pentobarbital. Lungs were perfused with PBS, excised, minced with a blade into small pieces and digested with 2 mg/ml collagenase Type III (Worthington Biochemical, Lakewood, NJ) and 0.1% DNase (Roche, Mannheim, Germany) at 37°C. Cells were separated from cell debris and erythrocytes via a Lympholyte M gradient (Cedarlane Labs, Burlington, NC). CD11c^+^ cells were positively magnetically sorted according to manufacturer’s instructions (MACS, Miltenyi Biotec, Bergisch Gladbach, Germany). Enriched CD11c^+^ cells were cultivated overnight at a density of 2–4×10^5^ cells per well on collagen-coated glass slides (Advanced BioMatrix, San Diego, CA) in a 24-well cell culture dish before motility analysis. For phagocytosis assays cells were used immediately without prior cultivation and were treated as described below.

### Phagocytosis Assay

BMDC or total lung CD11c^+^ cells (see above) were allowed to adhere for 1 h at a density of 2×10^5^ cells per well of a 96-well plate. Cells were stimulated with neuropeptides or water for 5 min. The following final neuropeptide concentrations were used: VIP with 0.1 nM, SP with 1 µM, and CGRP with 1 nM (all Phoenix pharmaceuticals, Burlingame, CA). Afterwards, *E. coli* pHrodo particles (Invitrogen, Darmstadt, Germany) were applied for 2 h. The particles are fluorescent at 592 nm under the acidic conditions of the phagolysosome thereby only marking cells that take up particles. As a negative control phagocytosis was inhibited with cytochalasin D (Sigma, München, Germany) in cells of control wells 30 min before particle application. Images were acquired with an LSM 510 META confocal microscope (Zeiss, Jena, Germany), using a plan neofluar 10×/NA0.3 objective (electronic zoom 3) and a 560–615 band pass filter. Particles were excited with a 543 nm 1 mW Helium Neon laser at 6% output. To allow comparison of pictures from different samples, detector gain and offset conditions were kept constant. The transmission signal was also collected to allow visualization of all cells in a well. As an internal standard in each picture a cell was set to the center of the imaging area and the focus plane was adjusted to allow comparison of similar focus layers between different wells.

All cells of an image were counted manually (transmission channel). The total area of fluorescent particles was determined with ImageJ software and was divided by the total cell number to calculate phagocytosis index (PI). For one experiment ten wells of each group (five of the Cytochalasin D control) were analyzed. Two images were taken from each well to determine the PI. Statistical analysis was performed using GraphPad Prism 4.03 and applying unpaired *t*-test.

### OVA-Sensitization and Allergen Challenge

Mice were sensitized according to a standard protocol [20] using 10 µg OVA (Grade VI; Sigma, München, Germany) adsorbed to 1.5 mg Al(OH)_3_ diluted in 0.9% NaCl on days 0, 14, and 21 via i.p. injection or sham-sensitized with 1.5 mg Al(OH)_3_ in 0.9% NaCl i.p., respectively. On day 27, all animals were exposed to 2% OVA aerosol in 0.9% NaCl for 20 min (“acute model”) and subsequently sacrificed with an i.p. administered overdose of pentobarbital to prepare living lung sections for time-lapse imaging. To mimic a more severe situation of allergic airway inflammation (“prolonged model”), the protocol was prolonged by two additional OVA aerosol challenges on days 27 and 28 and a final challenge at day 35 [21]. Mice were also sacrificed subsequently after the final challenge to prepare living lung sections for time-lapse imaging.

### Whole-mount Microscopy of Mouse Airways

The main bronchi of control and OVA-challenged mice were isolated 24 h after the last challenge and stained as whole-mounts as described earlier [4]. The following primary antibodies were used: Cytokeratin rabbit anti-mouse (Abcam, Cambridge, UK), MHC-II rat anti-mouse (Biolegend, San Diego, CA), F4/80 rat anti-mouse (Caltag, Buckingham, UK), CD68 rat anti-mouse (Acris, Herford, Germany), PGP9.5 rabbit anti-mouse, CGRP guinea pig anti-mouse (Acris, Herford, Germany), SP rat anti-mouse (Millipore, Schwalbach, Germany). Accordingly the following secondary antibodies were used: donkey anti-rabbit Cy3, donkey anti-rat Cy3 (Dianova, Hamburg, Germany), goat anti-rat Cy5 (Invitrogen) and donkey anti-guinea pig Cy5 (Millipore, Schwalbach, Germany). 3D-stacks were acquired with a LSM 510 META confocal microscope (Zeiss, Jena, Germany), using a C-Apochromat 40×/NA 1.2 water immersion objective (electronic zoom 0.7). Cy3 signal of PGP9.5^+^ nerves was excited with a 543 nm 1 mW Helium Neon laser at 10% output and detected using a 560–615 band pass filter. Cy5 fluorescence of neuropeptide signals were excited with a 633 nm 1 mW Helium Neon laser at 10% output and detected by applying a 650 long pass filter. To allow comparison of pictures from different samples, detector gain and offset conditions were kept similar. 3D *z*-stacks were acquired with a *z*-resolution of 1 µm, resulting in image volumes of 318 µm in *xy* and approximately 20 µm in *z*-direction. Maximum-intensity projections of *z*-stacks were generated with Imaris, analyzed with ImageJ and areas of PGP9.5^+^ and neuropeptide positive signals were determined, respectively. The percentage of neuropeptide positive signal of total nerve signal (PGP9.5^+^) per image was calculated. Per airway (main bronchus) six regions scattered along the whole length, beginning at the proximal part, were defined for imaging using branching points to the secondary bronchi for orientation. Between different airway samples always similar regions of these defined areas were analyzed. Statistical analysis was performed using GraphPad Prism 4.03 and applying unpaired *t*-test.

## Results

### CD11c^+^ Cells in Mouse Main Bronchi Show Characteristics of Dendritic Cells

**Figure 1:**
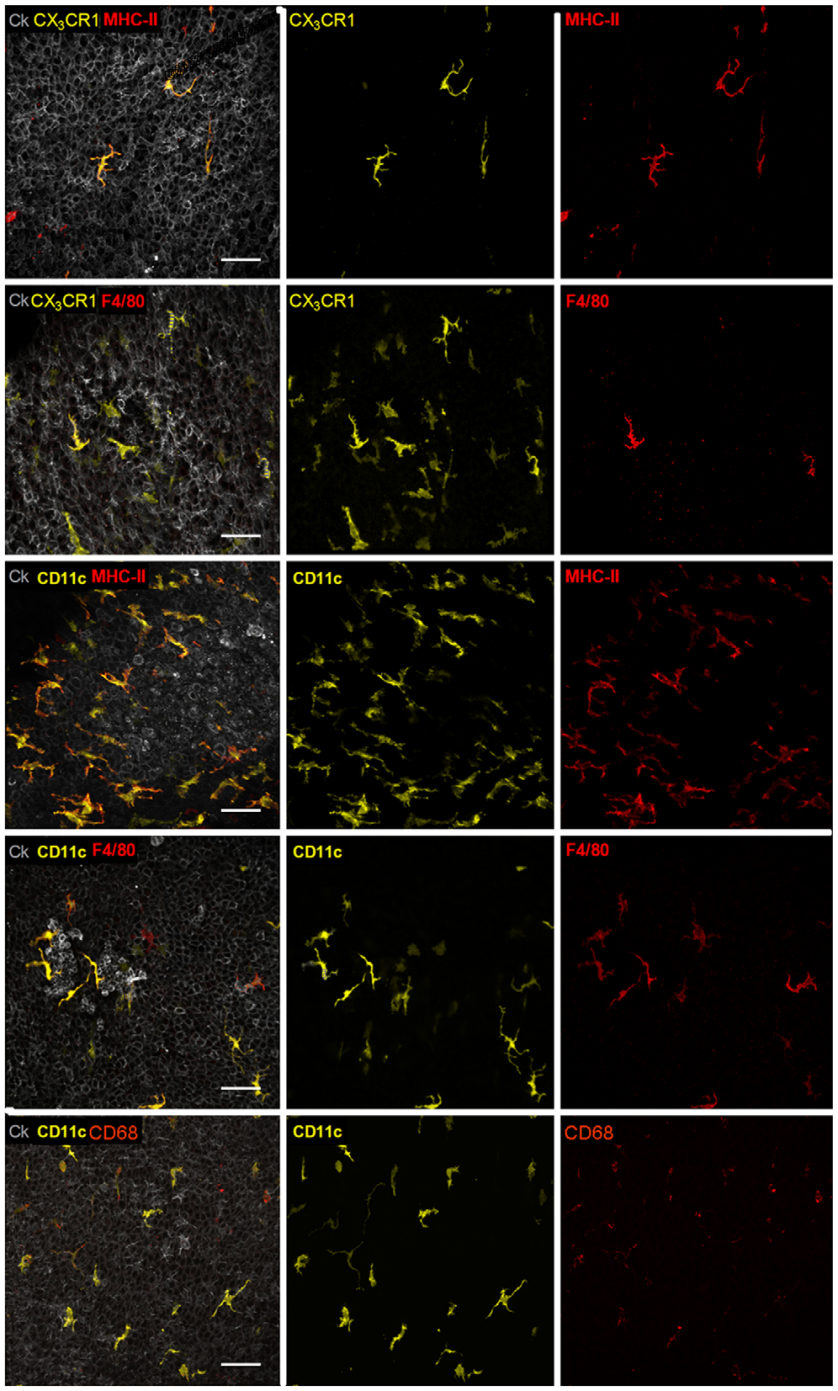
Characterization of CD11c^+^ cells in mouse main bronchi. Mouse airways of the CD11c-EYFP reporter mouse (CD11c in yellow) or of the CX_3_CR1^+/*gfp*^ transgenic mouse (CX_3_CR1 in yellow) were stained as whole-mounts against MHC-II (red), F4/80 (red) or CD68 (red) as indicated. Airway epithelium stained with cytokeratin (Ck) is shown in grey. Left column shows merges of all acquired channels. Other columns show according single channel images. Image projections were generated with Imaris (Bitplane) from raw confocal *z*-stacks. Scale: 40 µm.

**Figure 2:**
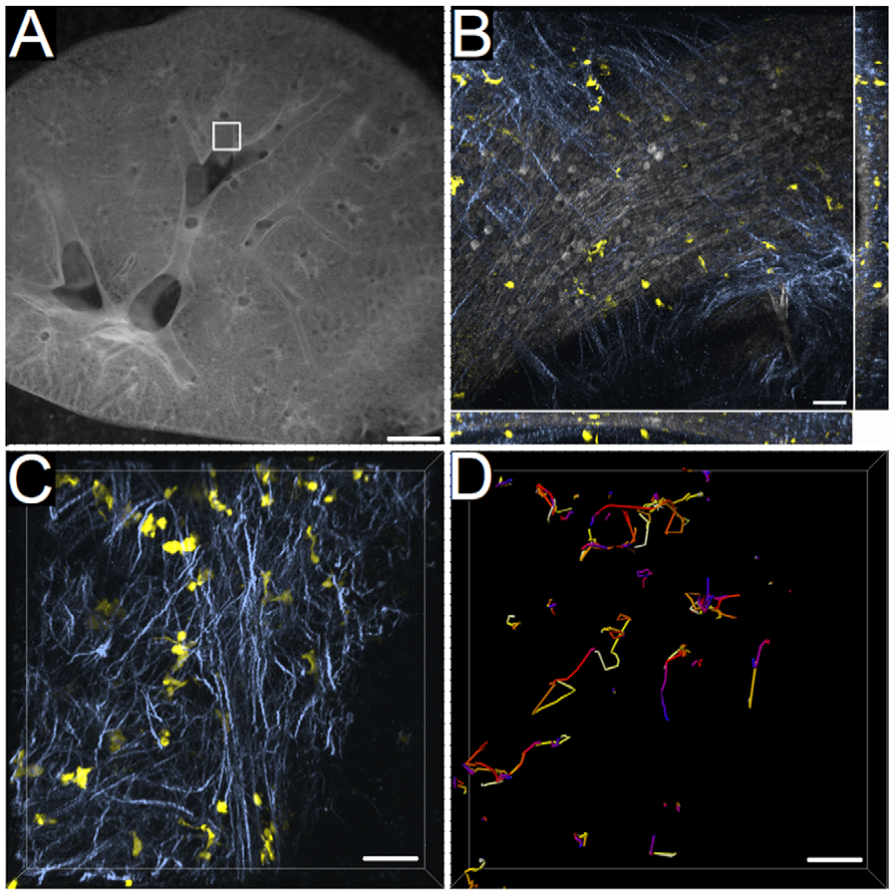
Analysis of DC motility in mouse main bronchi. Main bronchi of mouse living lung sections were analyzed by two-photon time-lapse microscopy. (A) 300 µm thick mouse living lung slice visualized with a stereomicroscope. Box indicates a typical region used for three-dimensional time-lapse microscopy. Scale: 1 mm. (B) Three-dimensional stack projection of DC in the airway epithelium. CD11c^+^ cells shown in yellow, SHG signal of airway collagen fibers in blue (two-photon excitation: 920 nm), autofluorescence of airway epithelium in grey (two-photon excitation: 820 nm). Side and bottom panel show side views of the stack in *yz* and *xz*-direction, respectively. Scale: 40 µm. (C) Example of a three-dimensional stack projection of a single analysis time point. CD11c^+^ cells of CD11c-EYFP transgenic mice are shown in yellow; SHG signal of collagen fibers of the airway in blue (two-photon excitation: 920 nm). Scale: 50 µm. (D) Color-coded tracks of DC during the total analysis time of 1 h image acquisition (resume pertains to C). Blue color code marks the start of imaging; white, the end of imaging. Scale: 50 µm.

In our study we focused on a CD11c^+^ immune cell population present in the distinct airway mucosal compartment [20]. These cells represent a heterogeneous population based on their morphology and movement behavior. In order to characterize these cells in mouse main bronchi, airway whole-mounts of CD11c-EYFP transgenic mice [19] were stained with a combination of markers discriminating between DC and other cell types like e.g. macrophages such as CD11c, major histocompatibility complex class II (MHC-II), CD68, and F4/80. Additionally airway whole-mounts of CX_3_CR1^+/*gfp*^ [22] transgenic mice were stained with the same marker combination. Because it appears that in both transgenic mice the same cell population is illuminated by their reporter fluorescence in the airway mucosa (fig. 1), we concluded that most CD11c^+^ cells in mouse main bronchus epithelium show markers described to be characteristic for lung DC (CD11c^+^ MHCII^+^ CX_3_CR1^+^ F4/80^+^ CD68^+^ phenotype) [23]–[27]. For our *ex vivo* imaging studies of DC in living mouse bronchi (fig. 2A) the CD11c-EYFP transgenic mouse was used [19]. In these transgenic mice all CD11c^+^ cells express the fluorochrome enhanced yellow fluorescent protein (EYFP) allowing an unaltered live cell imaging without any further labeling procedures. To make sure that only DC of the airway epithelium were captured for analysis additionally the SHG signal of collagen fibers was collected (fig. 2B, Video S1). These collagen fibers closely underlie the airway epithelium and therefore elegantly facilitate in-tissue orientation. For motility measurements in our study only cells co-localizing with these collagen fibers and being defined as airway mucosal DC were included (fig. 2C and D).

### Electrical Field Stimulation Enhances CD11c^+^ Cell Motility

**Figure 3:**
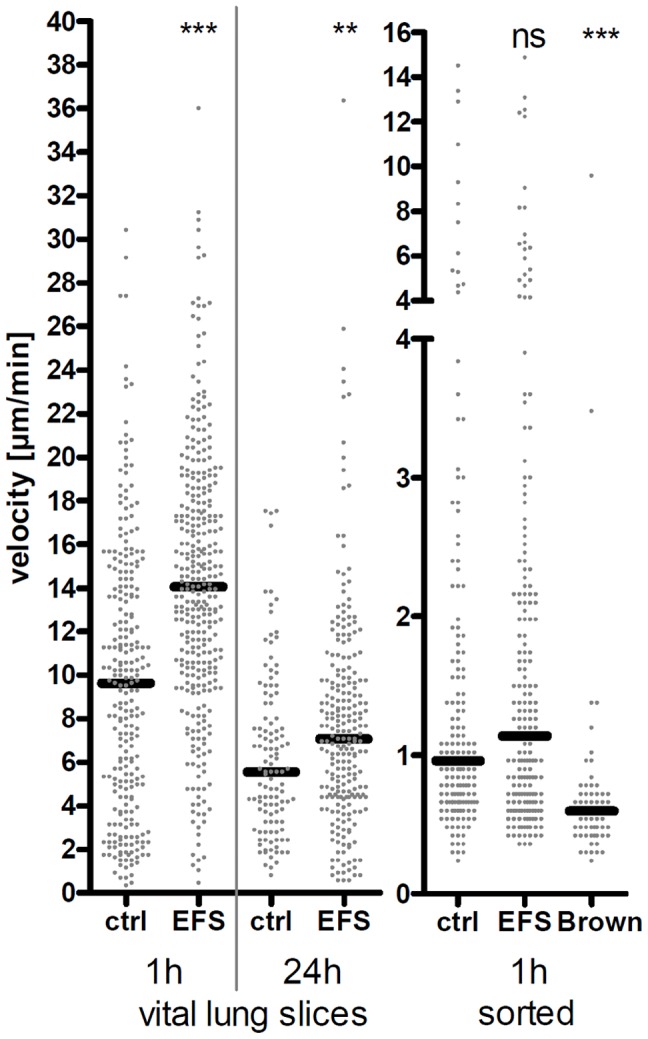
Motility of CD11c^+^ cells after electrical field stimulation. Mean velocities of lung CD11c^+^ cells were determined under untreated conditions (ctrl) or after different time-points (1 h, 24 h) of EFS. Each dot represents the mean velocity of an individual cell; black horizontal bars indicate the medians. Data of living lung slices show a pool of two independent experiments (left part). Mean velocities of *in vitro*-cultured magnetically sorted CD11c^+^ total lung cells (right part) were acquired from one experiment; Brownian Motion (Brown) was determined by analyzing non-motile paraformaldehyde-fixed cells. Mann Whitney statistical test; ns not significant; * *p*<0.05; ** *p*<0.01; *** *p*<0.001.

Initially we hypothesized that neuro-immune communication between immune cells e.g. DC and nerves occurs in the airways. In order to test this idea, we triggered the neuropeptide release in living lung slices using EFS [28] and determined DC velocity as the most obvious readout parameter to describe the motile attitude of DC. The neuropeptide release was controlled indirectly by monitoring bronchoconstriction of the airway in consequence of the axon reflex caused by neuropeptides [29]. For the first time we could show that either 1 h or 24 h after electrical field stimulation (EFS) DC motility in the main airways is significantly increased by 20–30% compared to non-stimulated controls (fig. 3). The difference between mean velocities of non-stimulated and stimulated DC is less at 24 h than at 1 h after EFS. This difference might occur because of minimal loss in vitality of the sample which is also indicated by the decreased (40%) baseline velocity of the control group 24 h after stimulation. To rule out that the electrical field itself directly influences DC motility, we cultured magnetically sorted whole lung CD11c^+^ cells on collagen matrix-covered glass slices at similar conditions, applied the same electrical field to these cells, and determined their motilities. The right part of figure 3 shows that there is no difference in motility between stimulated and non-stimulated cultured CD11c^+^ cells. Since baseline cell motility was decreased under culture conditions compared to living lung slices, the Brownian motion of PFA-fixed cultured cells was also determined. The mean velocity of cells showing only Brownian motion is significantly lower compared to control, indicating a proper motion of cultured whole lung CD11c^+^ cells under these experimental conditions. Data in fig. 3 confirmed the assumption that a nerve-derived transmitter is necessary to induce a change in DC motility after EFS.

### Neuropeptide Treatment Modulates Motility of Lung CD11c^+^ Cells

**Figure 4:**
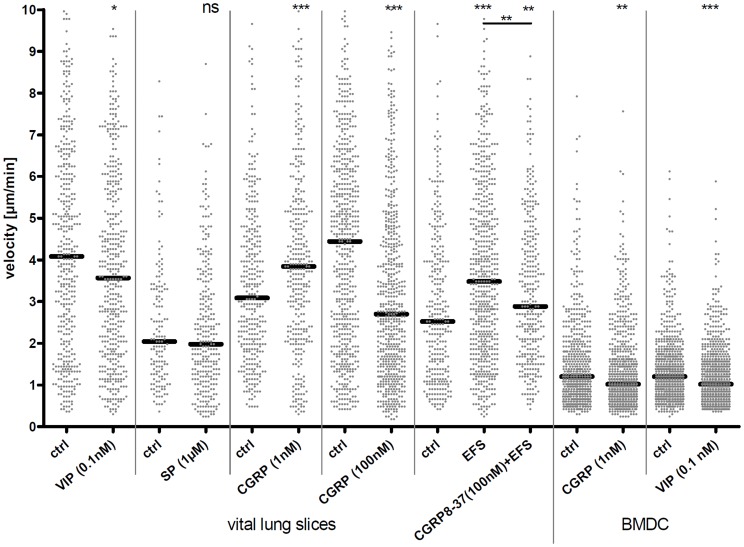
Motility of neuropeptide-treated CD11c^+^ cells. Mean velocities of lung CD11c^+^ cells and airway DC five minutes after neuropeptide treatment. Groups as indicated. For antagonistic treatment, slices were pre-treated with CGRP_8–37_ five minutes prior to EFS and analyzed 1 h after EFS. Each dot represents the mean velocity of an individual cell; black horizontal bars indicate the medians. Data of living lung slices and *in vitro*-generated BMDC show a pool of three independent experiments for each group. Mann Whitney statistical test; ns not significant; * *p*<0.05; ** *p*<0.01; *** *p*<0.001.

To further confirm the assumption that DC motility can be influenced by nerve-derived factors, we tried to pinpoint possible candidates. Others already found that DC migration could be modulated by neuropeptides *in vitro*. For our analyses we used neuropeptide concentrations that showed optimal responses in experiments performed by Dunzendorfer *et al.* [30]. Data in fig. 4 show velocities of DC in main bronchi of living lung slices after neuropeptide treatment. Treatment of lung slices with 0.1 nM VIP or 100 nM CGRP resulted in decreased airway DC motility, whereas 1 nM CGRP lead to an increased motility. 1 µM SP had no influence on airway DC motility. To further confirm that CGRP specifically mediates enhanced motility induced by EFS, living lung slices were pre-treated with a CGRP receptor antagonist and were then electrically stimulated (fig. 4). With 100 nM CGRP_8–37_ antagonistic treatment of living lung slices prior to EFS a partial reduction of EFS-induced increased DC motility could be achieved. *In vitro*-generated bone marrow-derived DC (BMDC) were also treated with neuropeptides and their motilities were determined (fig. 4). After applying 0.1 nM VIP DC also showed decreased motility as in living lung slices. When cells were stimulated with 1 nM CGRP DC slowed down which is in contrast to the results gained in living lung slices. After stimulating living lung sections with a higher concentration of CGRP (100 nM) DC slowed down as well.

### Neuropeptides Decrease Phagocytotic Capacity of Bone Marrow-derived Dendritic Cells and Lung CD11c^+^ Cells

Next we tested whether neuropeptides can also influence other characteristics of DC/CD11c^+^ cells. A crucial feature of antigen-presenting cells is their phagocytotic activity. Thus an assay was performed that specifically allowed determination of the phagocytotic capacity of CD11c^+^ cells. Therefore fluorescent *E. coli* particles were offered to BMDC or whole lung magnetically sorted CD11c^+^ cells *in vitro*. To measure only *E. coli* particles that were taken up by cells, pHrodo particles were employed that are fluorescent at the definite pH present in the phagolysosome.

**Figure 5:**
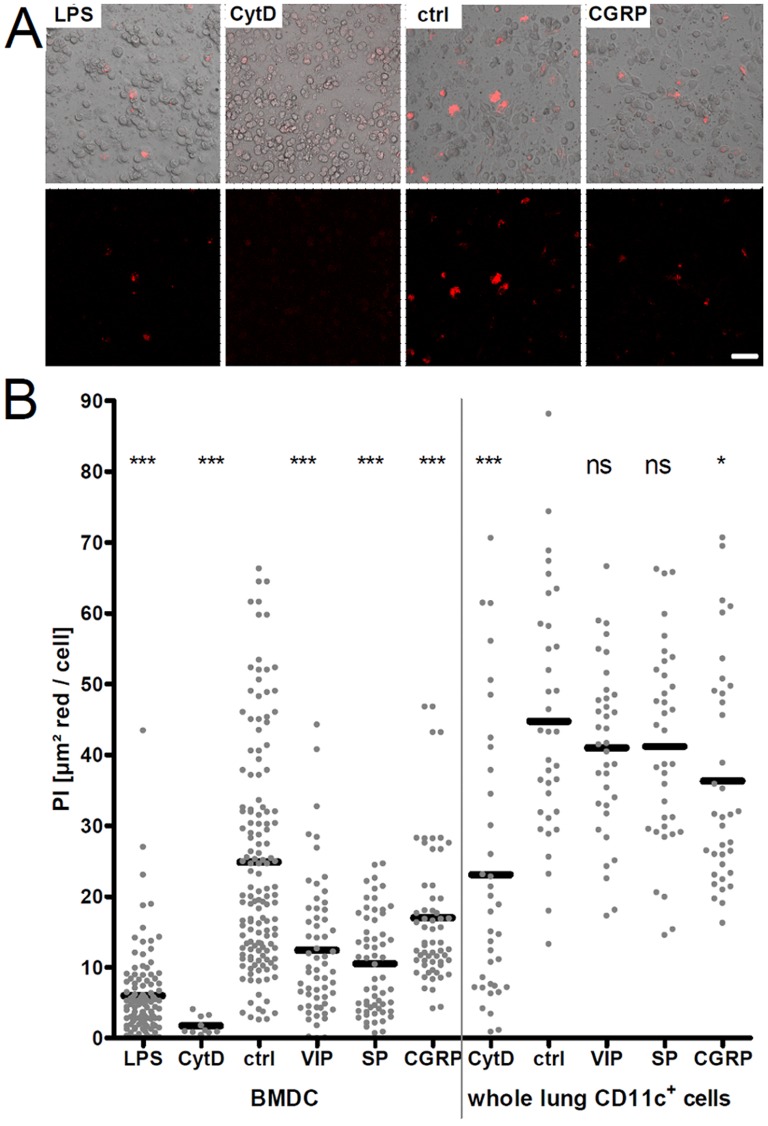
Phagocytosis capacity of CD11c^+^ cells after neuropeptide treatment. (A) Representative confocal images of *in vitro*-cultured BMDC after different treatments as indicated. Upper panel shows a merge of red fluorescence of phagocytosed *E. coli* pHrodo particles and transmitted light channel to visualize all cells. Lower panel shows only the red fluorescence according to images of the upper panel. Lower panel images were used to determine the total area of red fluorescence in each image with ImageJ software. Scale: 40 µm. (B) Graph of phagocytosis indices (PI) of BMDC and magnetically sorted total lung CD11c^+^ cells. Cells were neuropeptide or control treated for five minutes. *E. coli* pHrodo particles were then applied for 2 h. Beforehand a fraction of cells was triggered over night with LPS (three independent experiments). To inhibit phagocytosis, another fraction of cells was treated with Cytochalasin D (one experiment for BMDC, two experiments for whole lung CD11c^+^ cells). Neuropeptide-treated groups (0.1 nM VIP, 1 µM SP, 1 nM CGRP) were compared to a water-treated group (ctrl) (four independent experiments for BMDC and two independent experiments for whole lung CD11c^+^ cells). Each dot represents PI of one image. Black horizontal bars indicate the means. Per well two images were acquired. Unpaired statistical *t*-test; ns not significant; * *p*<0.05; ** *p*<0.01; *** *p*<0.001.

The phagocytotic index (PI) of cells treated with the phagocytosis inhibitor Cytochalasin D is significantly lower compared to the control-treated group (fig. 5). This shows a specific phagocytotic uptake of *E. coli* pHrodo particles by these cells. Additionally, the PI of BMDC matured by LPS treatment was compared to the PI of control cells. As expected the PI of matured cells is lower than of naïve cells. Furthermore, the morphology of the Cytochalasin D-treated group is altered. These cells exhibit a round cell shape, due to the inhibiting toxic effect of Cytochalasin D directly affecting the actin cytoskeleton.

Interestingly, the phagocytosis indices of BMDC treated with the neuropeptides VIP, SP or CGRP are significantly lower than of the control-treated cells indicating a decreased phagocytosis after neuropeptide trigger. Similar tendencies could be observed when treating whole lung magnetically sorted CD11c^+^ cells with neuropeptides although not reaching statistical significance in cases of VIP and SP.

### Altered Neuropeptide Levels in Mouse Main Bronchi in an Acute and Prolonged Model of Asthma

**Figure 6:**
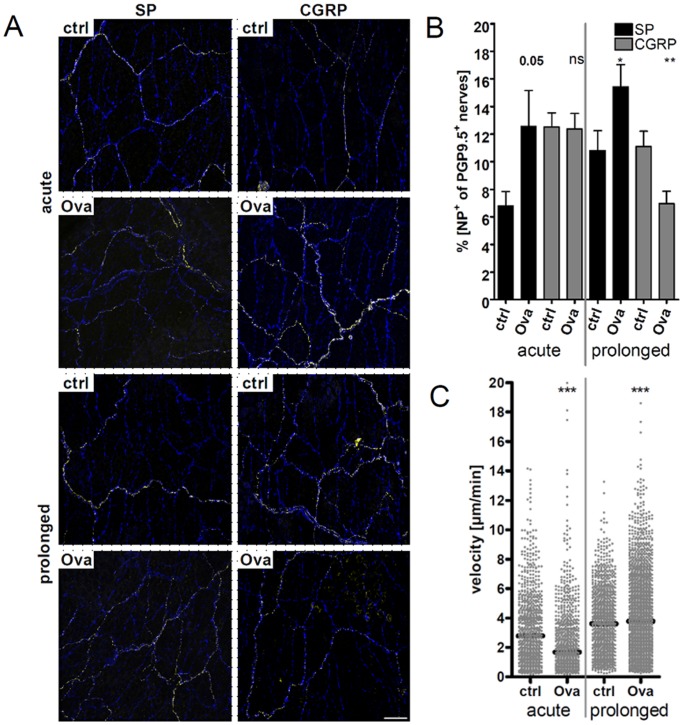
Determination of neuropeptide amounts and of DC motility in mouse airways in an acute and prolonged model of allergic airway inflammation. (A) Representative confocal *z*-stack projections of mouse airways stained as whole-mounts against the pan-neuronal marker PGP9.5 (blue) and neuropeptides SP or CGRP (yellow), respectively. Airways of control-treated and asthmatic mice were analyzed (acute model: i.p. booster on days 14 and 21, challenge and analysis on day 27; prolonged protocol: i.p. booster on days 14 and 21, two additional challenges on days 27 and 28, final challenge on day 35, analysis day 36). The total area of fluorescence signals of maximum intensity projections of *z*-stack images was determined by using Imaris and ImageJ software. (B) The total area of neuropeptide fluorescence signal (SP or CGRP signal) was set into relation to the area of all nerves (PGP9.5 signal). This ratio was determined for six regions of each airway. Per group three mice donating one airway each were analyzed. Unpaired statistical *t*-test; outliers were determined by Grubb’s test; ns not significant; * *p*<0.05; ** *p*<0.01; *** *p*<0.001. Scale: 40 µm. (C) Mean velocities of airway DC were determined both, in an acute OVA asthma model and in a model using a prolonged protocol and in their respective control groups (ctrl) as indicated. Each dot represents the mean velocity of an individual cell; black horizontal bars indicate the medians. Data of living lung slices show a pool of three (acute) to four (prolonged) independent experiments. Mann-Whitney statistical test; ns not significant; * *p*<0.05; ** *p*<0.01; *** *p*<0.001.

To investigate whether different neuropeptide levels are present in the microenvironment surrounding airway mucosal DC under pathogenic conditions, the amount of neuropeptide-expressing nerves was determined. It has been described that in the different stages of asthma, the expression of neuropeptides is altered [13], [15], [31]. First, percentages of neuropeptide-containing nerves were analyzed and compared in airways of mice subjected to an acute or prolonged model of allergic airway inflammation (fig. 6A). In the acute model, the SP content of airway nerves was increased in diseased mice compared to healthy controls (fig. 6B). The amount of CGRP within PGP9.5 positive fibers remained unaltered in the acute model. The amount of SP was also increased in the prolonged model whereas CGRP-amounts were decreased. Unfortunately, antibodies against VIP gave only faint signals; hence it was not possible to determine the VIP-content of nerves in the airways.

### DC Motility is Altered in Both the Acute and Prolonged Models of Asthma

Next we investigated whether altered neuropeptide expression (fig. 6C) during different stages of allergic airway inflammation could be responsible for an altered DC motility *in vivo*, too. Therefore the velocities of airway DC in both the acute and prolonged asthma model were compared with controls. In the classical acute model, airway DC showed decreased motilities (fig. 6C) compared to controls and at the same time higher amounts of neuropeptide-containing nerves (fig. 6B). Conversely, the airway DC in the prolonged model showed increased motilities compared to controls (fig. 6C) and at the same time higher SP- and lower CGRP-containing airway nerves.

## Discussion

The present study investigated neuro-immune interactions in the mouse main airways. A morphologically distinct CD11c^+^ MHC-II^+^ CX_3_CR1^+^ F4/80^+^ DC population present in the mucosal airway epithelium compartment was characterized and analyzed (fig. 1 and [23], [24]). We focused on the influence of neuropeptides on the behavior of this DC population *in situ* since DC express receptors for neuropeptides [1]–[3], [9]. Using two-photon time-lapse imaging we show for the first time the dynamics of airway mucosal DC under the influence of neuropeptides in living airway tissue. Neuropeptides can be released after several external stimuli as well as under endogenous inflammatory conditions. Under experimental conditions the release of endogenous sensory neuropeptides can be triggered electrically. EFS leads to an ubiquitous neuropeptide release through N type voltage-gated calcium channels in nerve endings [28]. Triggering living lung slices with an electrical field can mimic the neuropeptide release occurring during allergic airway inflammation. Our experiments show that the electrical stimulus led to an increased DC motility. This gave us the first hint that the altered DC behavior could be neurally mediated. Control experiments with cultured whole lung CD11c^+^ cells indicated that the electrical field by itself had no influence on DC motility. Although it is possible that in this cell preparation from the whole lung besides DC also other CD11c^+^ cell types (e.g. macrophages) are present. These cells could be non-responders to the electrical stimulus and thereby influence the motility readout. To further investigate whether neuropeptides could influence DC motility and to identify responsible candidates, synthetic neuropeptides were added specifically. As previous studies already identified neuropeptides that influenced *in vitro* chemotaxis of blood-derived DC, we used similar neuropeptide concentrations in our *ex vivo* experiments although we used different cell types [30].

We could show that indirect neuropeptide release by EFS as well as direct application of the neuropeptide CGRP (1 nM) could increase the motility of mouse airway DC in living lung slices. An opposite effect could be achieved with a higher CGRP concentration (100 nM) and after application of 0.1 nM VIP. SP had no influence on DC motility in this system and others also reported only a mild effect of SP on chemotaxis of naïve DC *in vitro* [30]. We gained the strongest evidence for an EFS-provoked neuropeptide release with subsequent influence on airway DC behavior by pre-incubation of living lung slices with the CGRP receptor antagonist CGRP_8–37_. Increased DC motility caused by EFS could specifically be blocked by CGRP_8–37_ pre-treatment, showing that CGRP is important to stimulate DC motility in this system. However, since the EFS effect could not be fully blocked by CGRP_8–37_ it appears that other neuropeptides besides CGRP are also responsible for modulating DC motility. In addition to airway DC we could also observe motility alterations of BMDC by neuropeptides. Data suggest that DC of different origin can respond to neuropeptides in order to arrest at sites of distinct neuropeptide concentrations or to accelerate their motility to carry out their proper function [30].

An equal neuropeptide concentration of CGRP was used for living lung slices and BMDC and surprisingly gave opposite results. Whereas 1 nM CGRP in lung slices resulted in increased DC motility the same concentration results in decreased motility of bone BMDC under the same experimental conditions. We assumed that the final available concentration of CGRP that reaches the cell is higher in the *in vitro* situation because cells are directly exposed to the neuropeptide-containing medium compared to cells in the lung slice that are covered by the airway epithelial barrier. Furthermore, tissue cells contain and release neuropeptide-degrading enzymes like the neutral endopeptidase (NEP), which additionally can lead to a declined local neuropeptide concentration. To test this hypothesis a higher CGRP concentration (100 nM) was applied to living lung slices. Indeed the DC motility decreased comparable to the *in vitro* BMDC setting using 1 nM of CGRP. Others using the same CGRP concentration also found a less motile phenotype *in vitro* [30]. Another explanation for differing results could provide the observation that neuropeptides can exhibit a bi-phasic dose-response: while lower doses specifically affect high-affinity receptors, higher doses also trigger the low-affinity receptors. It has been shown that the different receptors for CGRP exhibit different affinities for CGRP. Calcitonin receptor-like receptor (CRLR) can match with different activity-modifying proteins (Ramp). CRLR with Ramp1 represents a highly affine receptor for CGRP while the combination with Ramp2 or Ramp3 has only a low CGRP affinity. The CRLR/Ramp 2 or Ramp3 receptor might also respond after a trigger with a high CGRP concentration. These latter receptor combinations have been reported to have an inhibitory effect on cell migration [25], [32]. These observations fit to the opposite effects gained in our analyses after different CGRP concentrations.

In the following experiments we investigated whether neuropeptides can alter another key feature of DC, too, namely antigen-capture. We offered *E. coli* particles to the cells as reference particles to test genuine phagocytosis which exclusively can be performed by phagocytes. Other approaches using labeled OVA antigen turned out to be rather inapplicable due to uptake via unspecific macropinocytosis. Interestingly, the phagocytotic capacity of BMDC and of whole lung CD11c^+^ cells could be impaired by neuropeptides. The effect of neuropeptides on whole lung CD11c^+^ cells was weaker compared to BMDC which might be due to a different purity of the used cells. Whereas *in vitro*-generated cells have a consistent DC phenotype [33], whole lung CD11c^+^ cells are a mixture of DC and also macrophages [23] that might not respond to neuropeptides in the same way DC do. Additionally after preparation of lung cells and magnetic sorting, cells are not completely naïve anymore which might also explain the less prominent effect of neuropeptides on whole lung CD11c^+^ cells than on BMDC. Concordantly, for another epithelial surface it was observed by Hosoi *et al.* [34] that CGRP has the potential to inhibit antigen presentation abilities of Langerhans cells in the skin. One possible explanation for the influence of neuropeptides on motility and phagocytosis behavior is their potential to alter intracellular cAMP levels in lung DC by binding its receptor [3]. Furthermore, Kalamidas *et al.* observed that increased cAMP levels can inhibit actin assembly in macrophages [35]. Actin reassembly is necessary for cell motility and phagocytosis processes. Our data fit in well to this cascade, assuming that high CGRP levels lead to high internal cAMP levels, in turn to less actin assembly and subsequently to less motility.

After analyzing *in vitro* and *ex vivo* data we wanted to transfer insights to an *in vivo* situation. To mimic allergic airway inflammation we employed the commonly used OVA-induced asthma model. OVA-induced allergic airway inflammation can reproduce paras found during allergic asthma, as there are eosinophilic airway inflammation, airway hyperresponsiveness, elevated IgE levels, and antigen-induced tissue remodeling. Thus, we chose two different models of OVA-induced allergic airway inflammation and determined the amount of neuropeptide-containing nerves in the same microenvironment in which airway mucosal DC are located. Determining the amount of neuropeptide-containing nerves in relation to all (PGP9.5^+^) nerves in the airway epithelium displayed more SP-containing nerves both in the classical acute model and also in the prolonged model of allergic airway inflammation compared to sham-treated controls. These observations are in concordance with data from others, who detected higher SP levels in acute asthma [13]–[15].

On the other hand the amount of CGRP differed between our two OVA asthma models. In the acute model no difference in the amount of CGRP-containing nerves could be observed. At the same time motility data for high concentrations of CGRP (100 nM) as well as DC in acute asthmatic mice showed decreased DC motility. Accordingly, DC motility increased when less CGRP (1 nM) was applied and also in the prolonged asthma model where less CGRP-containing nerves were present. Others also reported about accelerated chemotaxis of naïve DC under the influence of low CGRP and VIP concentrations [30]. A recent publication showed that under physiological conditions of a skin inflammatory model (contact hypersensitivity) CGRP inhibited dermal DC migration to the draining lymph nodes [36]. This fits in well with the finding of Dunzendorfer *et al.* [30] who suggest that a high CGRP concentration present at the nerve during inflammation leads to an arrest of DC at this site. This observation also fits to our finding of decreased airway DC motility in the acute OVA model and after application of relatively high CGRP concentrations. On the other hand DC motility was increased in the prolonged OVA model which could mean that DC migration to the draining lymph nodes is dominating at this stage of inflammation. Analogously lower CGRP concentrations were measured in airways of mice treated under the prolonged protocol. Furthermore, under these conditions CGRP receptor expression is transcriptionally down-regulated on DC [3].

Taken together, the misbalanced ratio between stimulatory (SP) and inhibitory (CGRP) neuropeptides in prolonged airway inflammation leads to a predominance of a stimulatory milieu in the airway mucosa. This microenvironment supports the stimulation of DC which contributes on the long run to the development of a chronic inflammation status. In this milieu phagocytosis by DC is inhibited and further DC maturation can be prevented, too [3]. This could be a “stand-by” status of the DC. This is biologically reasonable since sensory nerve stimulation is almost always associated with tissue damage and infection. A neuropeptide-induced arrest would increase the number local DC on sites of damage and the block of maturation would preserve their ability to take up antigen. Upon phagocytosis and dependent on further signals DC would either migrate to the local lymph nodes to induce an adequate T cell immune response or interact with local T cell in the lung to (re)stimulate antigen-specific T cells directly in the lung tissue [4]. Taken together, neuropeptides could serve as an additional alert signal optimizing adequate immune responses to pathogens, on sites of injury, or damage.

Our data suggest that different neuropeptide levels in different stages of allergic airway inflammation alter airway DC motility correlating with their momentarily required function. It also appeared that the ratio between neuropeptides seems to be more important for a functional physiological interplay than their absolute concentration. We could demonstrate that neuropeptides directly influence airway mucosal DC behavior in living bronchial tissue. Future studies still need to reveal how a neuropeptide-modulated migration links to the role of DC during acute and prolonged inflammation. A potential therapeutic approach may be the pre-treatment of DC with a combination of neuropeptides or their antagonists to diminish or even prevent the development of airway inflammation. In summary, we present data suggesting a crucial role of neuropeptides in different stages of allergic airway inflammation to fine-tune airway DC behavior.

## Supporting Information

Video S1 **Airway mucosal dendritic cells in main bronchi of mouse living lung sections analyzed by two-photon microscopy.** Representative three-dimensional time-lapse movie of motile DC in the airway epithelium 1 h after EFS. CD11c^+^ cells of CD11c-EYFP transgenic mice are shown in yellow, SHG signal of airway collagen fibers in blue (two-photon excitation: 920 nm). Total analysis time of image acquisition: 55 min.
(MOV)
